# Pan-Canadian Electronic Medical Record Diagnostic and Unstructured Text Data for Capturing PTSD: Retrospective Observational Study

**DOI:** 10.2196/41312

**Published:** 2022-12-13

**Authors:** Leanne Kosowan, Alexander Singer, Farhana Zulkernine, Hasan Zafari, Marcello Nesca, Dhasni Muthumuni

**Affiliations:** 1 Department of Family Medicine Rady Faculty of Health Sciences University of Manitoba Winnipeg, MB Canada; 2 School of Computing Queen's University Kingston, ON Canada; 3 Manitoba Centre for Health Policy University of Manitoba Winnipeg, MB Canada; 4 Department of Psychiatry Rady Faculty of Health Sciences University of Manitoba Winnipeg, MB Canada

**Keywords:** electronic health records, EHR, natural language processing, NLP, medical informatics, primary health care, stress disorders, posttraumatic, posttraumatic stress disorder, PTSD

## Abstract

**Background:**

The availability of electronic medical record (EMR) free-text data for research varies. However, access to short diagnostic text fields is more widely available.

**Objective:**

This study assesses agreement between free-text and short diagnostic text data from primary care EMR for identification of posttraumatic stress disorder (PTSD).

**Methods:**

This retrospective cross-sectional study used EMR data from a pan-Canadian repository representing 1574 primary care providers at 265 clinics using 11 EMR vendors. Medical record review using free text and short diagnostic text fields of the EMR produced reference standards for PTSD. Agreement was assessed with sensitivity, specificity, positive predictive value, negative predictive value, and accuracy.

**Results:**

Our reference set contained 327 patients with free text and short diagnostic text. Among these patients, agreement between free text and short diagnostic text had an accuracy of 93.6% (CI 90.4%-96.0%). In a single Canadian province, case definitions 1 and 4 had a sensitivity of 82.6% (CI 74.4%-89.0%) and specificity of 99.5% (CI 97.4%-100%). However, when the reference set was expanded to a pan-Canada reference (n=12,104 patients), case definition 4 had the strongest agreement (sensitivity: 91.1%, CI 90.1%-91.9%; specificity: 99.1%, CI 98.9%-99.3%).

**Conclusions:**

Inclusion of free-text encounter notes during medical record review did not lead to improved capture of PTSD cases, nor did it lead to significant changes in case definition agreement. Within this pan-Canadian database, jurisdictional differences in diagnostic codes and EMR structure suggested the need to supplement diagnostic codes with natural language processing to capture PTSD. When unavailable, short diagnostic text can supplement free-text data for reference set creation and case validation. Application of the PTSD case definition can inform PTSD prevalence and characteristics.

## Introduction

Primary care providers are typically the first point of contact for individuals within the health care system. Primary care services support patients throughout their health care experiences managing both acute and chronic conditions. Primary care electronic medical records (EMR) are a rich source of longitudinal patient data collected by health care providers throughout an individual’s health care experience. EMR data can identify clinical phenotypes, describe care pathways, and inform quality improvement initiatives [1,2]. EMR-derived data typically include information related to patient characteristics, diagnoses, prescribed medications, and biometrics. They may also include information on social history, allergies, and risk factors for diseases [3-7]. Given the breadth of information available within EMRs, their use for disease surveillance continues to grow.

Identification of complex medical conditions may require multiple data points. Structured data fields such as standardized diagnosis or medication codes, as well as unstructured free-text data within the EMR can be assessed to describe complex conditions. Unstructured free text in the EMR can describe the observations, assessment, and plan for patient care providing depth to what is available in structured data fields [8,9]. More specifically, unstructured and short-text fields describe the patient context, including sociodemographic, risk behaviors and allergies, patient experience and interactions with the provider, and rational for the health care decisions that were made, which can inform disease surveillance and research [8]. Text analytics and, more specifically, natural language processing (NLP) of text data in the EMR can identify symptoms and variable interactions across multiple tables within data holdings [9-15]. Mining text data from health records typically includes refining procedures and knowledge extraction, aggregation, abstraction, and summarization of EMR information to transform text data into actionable insights such as inform phenotyping, disease prognosis and management, and disease surveillance [9,16,17]. Free-text information is not always available for research due to the technical limitations of EMR data systems or analysis, as well as privacy and data protection restrictions [18]. Due to this limitation, previous studies have relied on small data sets or a small number of institutions, preventing evidence of transferability of the models [17]. Primary care EMR short diagnostic text fields, more widely available than free-text data, have been suggested as a method for supplementing diagnostic definitions when free-text is unavailable [15,19,20]. Supplementation of free-text data with short-text fields, matched with refined processes for annotation and classification can support the use of EMR data in research [17].

Posttraumatic stress disorder (PTSD) is a complex mental health disorder characterized by a constellation of distressing symptoms that occur after witnessing or experiencing a traumatic event [21,22]. PTSD involves intrusive thoughts, persistent avoidance, negative alterations in cognition and mood, and alterations in arousal and reactivity (eg, irritability, reduced concentration, and exaggerated startle response) due to trauma recollection, which occur for greater than 1 month and result in significant impairment for the individual [20,22-24]. PTSD is associated with an array of multimodal risk indicators suggesting no single factor can account for the large variance in PTSD symptoms [19,20]. When encountered in primary care, PTSD is associated with considerable functional impairment and health care utilization [24]. This complex set of symptoms, combined with an individual’s possible reluctance to seek help, infrequent patient-clinician interaction, and overlapping symptoms with other mental health conditions, makes PTSD difficult to accurately diagnose in primary care [20,22]. Identifying PTSD requires both depth and breadth to detail the patients’ experience and capture associated factors [19,20].

This study had two objectives, which are as follows: (1) to compare the quality of capture when using free-text data compared to short diagnostic text fields from primary care EMRs for the creation of a reference set for a complex condition such as PTSD, and (2) test possible PTSD case definitions using single-province and pan-Canadian EMR reference standards. This study assesses the performance of 4 PTSD case definitions against reference standards to assess improved agreement when structured data fields are supplemented with NLP of EMR short diagnostic phrases.

## Methods

### Overview

This retrospective cross-sectional study used EMR data extracted and processed by the Canadian Primary Care Sentinel Surveillance Network (CPCSSN). At the time of this study, there were 1574 consenting primary care providers (ie, family physicians, nurse practitioners, and community pediatricians) from 257 clinics representing 1,493,516 patients in 7 Canadian provinces (British Columbia, Alberta, Manitoba, Ontario, Quebec, Nova Scotia, and Newfoundland and Labrador) [3,7].

### Data Sources

The CPCSSN repository is a pan-Canadian data set that is updated semiannually from regional practice-based research networks. The data in the repository comprised deidentified EMR data from consenting primary care providers that use 11 different EMR systems across Canada. Extracted EMR data are cleaned and standardized to map prescribed medications to Anatomical Therapeutic Chemical classification codes, laboratory tests to Logical Observation Identifiers Names and Codes, and medical diagnoses to International Classification of Disease, ninth edition, clinical modification (ICD-9-CM) codes. The CPCSSN repository also contains unstructured data in the form of short diagnostic text fields related to diagnoses, medication instructions, allergies, and social and behavioral risk factors. Additionally, some regional networks, such as the Manitoba Primary Care Research Network (MaPCReN), also extract free-text encounter notes that go through a deidentification algorithm to anonymize the data. Encounter notes are narrative entries created by primary care providers, typically structured in the problem-oriented medical record format [8]. MaPCReN represents 266 consenting primary care providers in 48 clinics in Manitoba, Canada. This study accessed a CPCSSN data set comprised of structured and short diagnostic text fields, and a MaPCReN data set containing structured, short diagnostic text fields, and free-text encounter notes.

### Manitoba Primary Care Patients

The MaPCReN database includes 289,523 patients, of which 154,118 (52.23%) were considered active because they had seen a primary care provider participating in MaPCReN in the prior 2 years (between January 1, 2017, and December 31, 2019) [25]. In addition to structured and short diagnostic text data available for all patients, 19.6% (56,795/289,523) of the patients have free-text encounter notes available in the MaPCReN repository (2,125,961 encounter notes). Two medical students conducted a complete review of the medical records of a subset of patients from the MaPCReN repository. The reviewers were instructed to use the criteria from the Diagnostic and Statistics Manual of Mental Disorders, Fifth Edition [26] or specific documentation to indicate whether a patient was diagnosed with PTSD. A data extraction form was developed to capture patients living with PTSD and related signs or symptoms (Multimedia Appendix 1).

**Figure 1:**
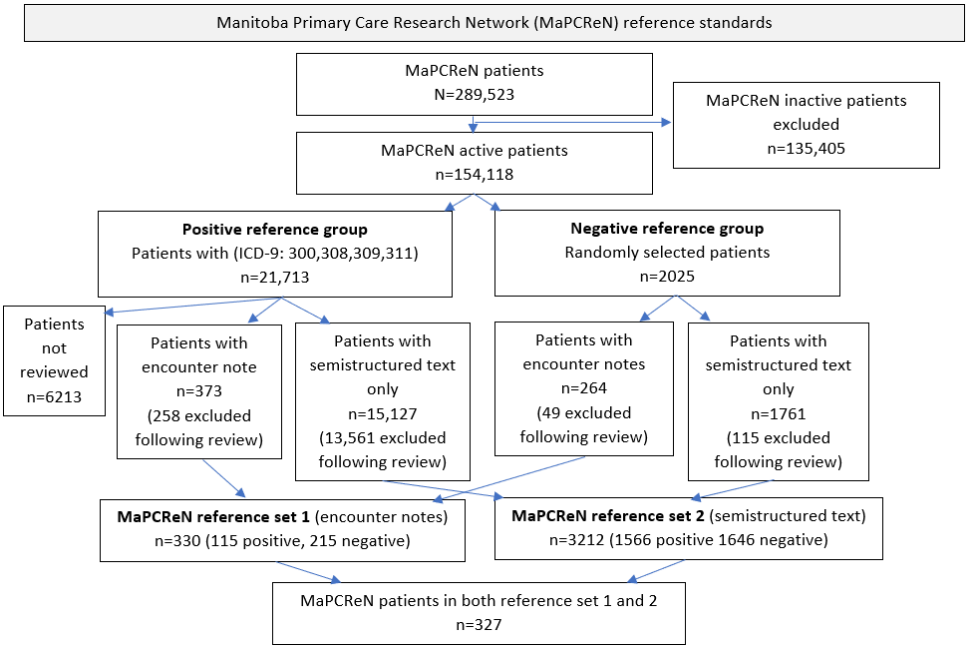
Flow diagram for creation of posttraumatic stress disorder reference standard in the Manitoba Primary Care Research Network.

To create the subset for medical record review, we identified 21,713 patients with one more of the following ICD-9-CM codes in the health condition table of the EMR starting 300 (anxiety), 308 (acute reaction to stress), 309 (adjustment reaction), or 311 (depression). A total of 373 patients had a complete record reviewed by 2 students. Medical record review without free text was also completed by 2 medical students for 15,127 (69.67%) of these 21,713 patients to create positive reference sets. To identify patients without PTSD (negative reference set), patients were randomly selected for review by 2 medical students. In the negative reference set, 264/2025 (13.0%) patients had full medical records review (including free text), and 1761/2025 (87.0%) patients were reviewed without free-text encounter notes. Patients were labeled as “PTSD,” “possible PTSD,” or “no PTSD” in the data extraction form (Multimedia Appendix 1). Any discrepancies were reviewed by a family physician clinician researcher (AS). The final reference set included patients who were considered “PTSD” or “no PTSD” and excluded patients with “possible PTSD.” This process created the following two MaPCReN reference standards: (1) a total of 330 patients (n=115, 34.8% positive and n=215, 65.2% negative) had full medical record review including free-text data, and (2) a total of 3212 patients (n=1566, 48.75% positive and n=1646, 51.25% negative) had medical record review without free-text data. There were 327 patients who were included in both MaPCReN reference sets (Figure 1) [20].

### Pan-Canadian Primary Care Patients

**Figure 2:**
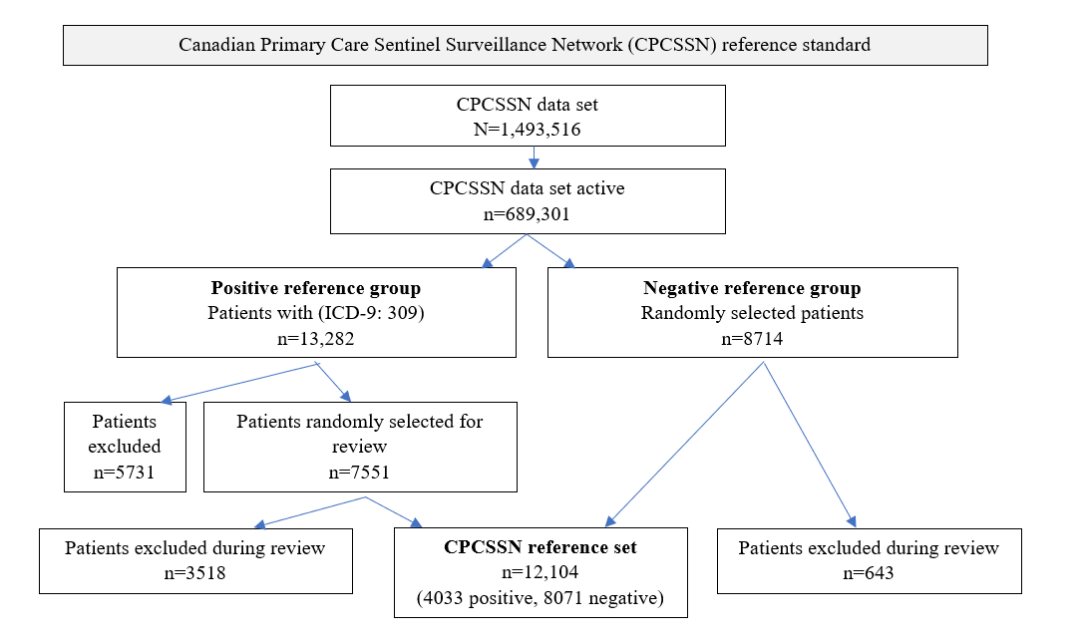
Flow diagram for creation of posttraumatic stress disorder reference standard in the Canadian Primary Care Sentinel Surveillance Network.

From the CPCSSN repository, a subset of patient records was extracted for medical record review to create a pan-Canadian reference set for PTSD. The CPCSSN repository contains EMR data for 1,493,516 patients, of which 689,301 (46.15%) were considered active because they had an appointment within the previous 2 years [25]. Within CPCSSN, there is no free-text encounter note data available. Medical record review was performed by 12 medical students using short diagnostic text fields. In total, there were 6 cohorts of ~2700 randomly selected records, each reviewed by 2 medical students for a total of 16,265 records reviewed. We included patients from each of the 7 participating provinces. There were 13,282 patients with an ICD-9-CM code (309, adjustment reaction), of which 7551 (56.85%) were randomly selected for medical record review. Moreover, there were 8714 patients randomly selected for creation of the negative reference set. We used the same data extract table and process as conducted for the MaPCReN reference set. Discrepancies were reviewed by a family physician (AS). There were 3518/7551 (46.6%) who were excluded due to poor interrater agreement or being classified as “possible PTSD.” Our final reference set had 12,104 patients (n=4033, 33.32% positive and n=8071, 66.68% negative; Figure 2).

### Case Definitions

Four case definitions for PTSD were developed by consensus discussion and evidence review by a research team including clinicians and researchers. Case definitions included ICD-9-CM and Anatomical Therapeutic Chemical codes from the health condition, billing, encounter diagnosis, and medication tables of CPCSSN (Table 1). The ICD-9-CM code for PTSD is 309.81; however, some providers use a less specific ICD-9-CM code 309 (adjustment reaction) because of billing rules in some justifications (ie, Ontario) which require that only the first 3 digits of the ICD-9-CM code be entered. Additionally, during medical record review, medical students found that patients with a diagnostic text entry for “PTSD” also had the following ICD-9-CM codes associated with that encounter: 300 (anxiety), 308 (acute reaction to stress), 309 (adjustment reaction), or 311 (depressive disorder). Medical student reviewers were instructed to create a list of spelling mistakes, abbreviations, and phrases that were recorded by primary care providers to identify PTSD in the short diagnostic text field (Multimedia Appendix 2). These codes and list were incorporated into data preprocessing stages prior to applying the case definitions (Table 1).

**Table 1:** Posttraumatic stress disorder (PTSD) test case definitions.

Case definition 1	Case definition 2	Case definition 3	Case definition 4
≥1 health condition, billing, or encounter diagnosis for ICD-9-CM^a^ 309.81	≥1 health condition for ICD-9-CM 309.81 OR ≥2 billing, encounter diagnosis for ICD-9-CM 309.81 separated by at least 1 week	≥1 health condition for ICD-9-CM 309.81 OR ≥1 billing, encounter diagnosis for ICD-9-CM 309.81 AND PTSD medication (ATC^b^ code starting with N05 or N06) OR ≥2 billing, encounter diagnosis for ICD-9-CM 309.81 separated by at least 1 week	≥1 health condition, billing, or encounter diagnosis for ICD-9-CM 309.81 OR ≥1 health condition, billing, or encounter diagnosis for ICD-9-CM starting with 290-316 AND PTSD recorded as the diagnosis name by the provider (Multimedia Appendix 2)

^a^ICD-9-CM: International Classification of Disease, ninth edition, clinical modification.

^b^ATC: anatomical therapeutic chemical.

### Preprocessing Steps

Primary care EMR data are collected for clinical purposes and therefore often include domain-specific language and acronyms as well as spelling and typographical errors. To prepare the data for validation (ie, capture in case definition 4), we removed stop words, removed special characters, and adjusted capitalization in the short diagnostic text fields of the EMR. Short diagnostic text fields document diagnosis name and reasons for the encounter. During medical record review, medical student reviewers recorded PTSD acronyms and spelling errors that were later converted into “PTSD” prior to applying the case definition (Multimedia Appendix 2).

### Statistical Analyses

We compared the agreement of EMR free-text encounter notes and EMR short diagnostic text fields using a 2x2 contingency table and the following metrics: sensitivity, specificity, positive predictive value (PPV), negative predictive value (NPV), and overall accuracy. Further, we assessed agreement between the PTSD case definitions and each of the 3 reference sets (MaPCReN free text, MaPCReN short diagnostic text, and CPCSSN) with sensitivity, specificity, PPV, NPV, and overall accuracy. The equations for these metrics are presented below:

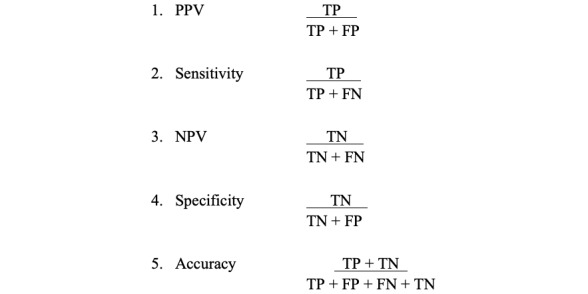



Using the PTSD case definitions, the prevalence and 95% confidence limits were computed using an exact binomial test to estimate prevalence of PTSD in a pan-Canadian data set. Statistical analyses were conducted using SAS V9.4 (SAS Institute).

### Ethics Approval

Ethical approval for this study was obtained from the Health Research Ethics Board at the University of Manitoba, approval number HS21053(2017:257).

## Results

### Manitoba Primary Care Patients

There were 154,118 patients in MaPCReN who attended an appointment with a participating provider between January 1, 2017, and December 31, 2019. There were 330 patients in MaPCReN reference set 1 (free-text data), and 3212 patients in MaPCReN reference set 2 (short diagnostic text). There were 327 patients who were included in both reference sets. There was a strong agreement between free-text and short diagnostic text reference sets with an overall accuracy of 93.6% (CI 90.4%-96.0%). There were 20 patients who had ongoing symptoms of PTSD documented in free-text EMR data (not an explicit PTSD diagnosis) that were not identified through review of short diagnostic text fields. Despite this, there was strong agreement between the 2 reference sets with a sensitivity of 82.5% (CI 74.2%-88.9%) and specificity of 99.5% (CI 97.4%-100%; Table 2).

Case definitions 1 and 4 performed similarly in both MaPCReN reference sets (Table 3). Reference set 1 had a sensitivity of 82.6% (CI 74.4%-89.0%), specificity of 99.5% (CI 97.4%-100%), PPV of 99.0% (CI 93.1%-99.9%), NPV of 91.5% (87.8%-94.1%), and accuracy of 93.6% (CI 90.4%-96.0%) for both case definitions. Similarly, reference set 2 had a sensitivity of 100% (CI 99.8%-100%), specificity of 98.4% (CI 97.7%-99.0%), PPV of 98.4 (CI 97.6%-98.9%), NPV of 100%, and accuracy of 99.2% (CI 98.8%-99.5%) for both case definitions. Within the MaPCReN repository, supplementation with NLP (case definition 4) did not capture any additional patients when compared to case definition 1, which focused only on diagnostic codes for PTSD (ICD-9-CM 309.81). Requiring a second billing code for PTSD (case definition 2) or a medication that may be used to treat PTSD (case definition 3) produced lower sensitivity (57.4%, CI 47.8%-66.6% and 79.1%, CI 70.6%-86.2%; Table 3).

**Table 2:** Agreement between Manitoba Primary Care Research Network (MaPCReN) reference set 1 (with encounter notes) and MaPCReN reference set 2 (with short diagnostic text fields only; N=327).

Performance metric^a^	Value (95% CI)
Accuracy	93.6 (90.4-96.0)
Sensitivity	82.5 (74.2-88.9)
Specificity	99.5 (97.4-100)
Positive predictive value	99.0 (93.0-99.9)
Negative predictive value	91.4 (87.7-94.0)

^a^Cell occurrence <5 required suppression of numbers in 2x2 contingency table.

**Table 3:** Agreement between patients captured using the posttraumatic stress disorder case definitions and the Manitoba Primary Care Research Network (MaPCReN) reference sets.

Case definitions		^a^TP (n)	^b^TN (n)	^c^FN (n)	^d^FP (n)	^e^SE (%, CI)	^f^SP (%, CI)	^g^PPV (%, CI)	^h^NPV (%, CI)	Accuracy (%, CI)	
**MaPCReN reference set 1 (with encounter notes; N=330)**											
	Case definition 1	95	214	*Suppressed*	<5	82.6 (74.4-89.0)	99.5 (97.4-100)	99.0 (93.1-99.9)	91.5 (87.8-94.1)	93.6 (90.4-96.0)	
	Case definition 2	66	214	*Suppressed*	<5	57.4 (47.8-66.6)	99.5 (97.4-100)	98.5 (90.3-99.8)	81.4 (77.9-84.4)	84.9 (80.5-88.5)	
	Case definition 3	91	214	*Suppressed*	<5	79.1 (70.6-86.2)	99.5 (97.4-100)	98.9 (92.8-99.9)	89.9 (86.2-92.7)	92.4 (89.0-95.0)	
	Case definition 4	95	214	*Suppressed*	<5	82.6 (74.4-89.0)	99.5 (97.4-100)	99.0 (93.1-99.9)	91.5 (87.8-94.1)	93.6 (90.4-96.0)	
**MaPCReN reference set 2 (no encounter notes; N=3212)**											
	Case definition 1	1566	1620	0	26	100 (99.8-100)	98.4 (97.7-99.0)	98.4 (97.6-98.9)	100	99.2 (98.8-99.5)	
	Case definition 2	1135	1640	431	6	72.5 (70.2-74.7)	99.6 (99.2-99.9)	99..5 (98.8-99.8)	79.2 (77.8-80.5)	86.4 (85.2-87.6)	
	Case definition 3	1469	1620	97	26	93.8 (92.5-95.0)	98.4 (97.7-99.0)	98.3 (97.5-98.8)	94.4 (93.2-95.3)	96.2 (95.5-96.8)	
	Case definition 4	1566	1620	0	26	100 (99.8-100)	98.4 (97.7-99.0)	98.4 (97.6-98.9)	100	99.2 (98.8-99.5)	

^a^TP: true positive.

^b^TN: true negative.

^c^FN: false negative.

^d^FP: false positive.

^e^SE: sensitivity.

^f^SP: specificity.

^g^PPV: positive predictive value.

^h^NPV: negative predictive value.

### Pan-Canadian Primary Care Patients

In the CPCSSN data set, case definition 4 had the strongest agreement with our reference set with a sensitivity of 91.1% (CI 90.1%-91.9%), specificity of 99.1% (CI 98.9%-99.3%), PPV of 98.1% (CI 97.6%-98.5%), NPV of 95.7% (CI 95.3%-96.1%), and accuracy of 96.4% (CI 96.1%-96.8%). In comparison, case definition 1 had a sensitivity of 72.3% (CI 70.9%-73.7%), specificity of 99.1% (CI 98.9%-99.3%), PPV of 97.6% (CI 97.0%-98.1%), NPV of 87.8% (CI 87.2%-88.3%), and accuracy of 90.2% (CI 89.7%-90.7%). The inclusion of multiple billing codes (case definition 2) or medications that can be used to treat PTSD (case definition 3) did not improve the agreement of the case definitions (Table 4).

When we apply each of the definitions to the CPCSSN data set of active patients, PTSD prevalence estimates suggest a range of 0.8% (CI 0.77%-0.81%; n=5565) with case definition 2 to 1.3% (CI 1.25%-1.31%; n=8913) with case definition 4. Case definition 1, which required at least one specific ICD-9-CM code 309.81, had a prevalence of 1.1% (CI 1.08%-1.13%; n=7718).

**Table 4:** Agreement between the posttraumatic stress disorder case definitions in the Canadian Primary Care Sentinel Surveillance Network and the reference data set (N=12,104).

Case definitions	TP^a^ (n)	TN^b^ (n)	FN^c^ (n)	FP^d^ (n)	SE^e^ (%, CI)	SP^f^ (%, CI)	PPV^g^ (%, CI)	NPV^h^ (%, CI)	Accuracy (%, CI)
1	2917	8000	1116	71	72.3 (70.9-73.7)	99.1 (98.9-99.3)	97.6 (97.0-98.1)	87.8 (87.2-88.3)	90.2 (89.7-90.7)
2	2502	8045	1531	26	62.0 (60.5-63.5)	99.7 (99.5-99.8)	99.0 (98.5-99.3)	84.0 (83.5-84.5)	87.1 (86.5-87.7)
3	2917	8004	1116	67	72.3 (70.9-73.7)	99.2 (99.0-99.4)	97.8 (97.2-98.2)	87.8 (87.2-88.3)	90.2 (89.7-90.8)
4	3672	8000	361	71	91.1 (90.1-91.9)	99.1 (98.9-99.3)	98.1 (97.6-98.5)	95.7 (95.3-96.1)	96.4 (96.1-96.8)

^a^TP: true positive.

^b^TN: true negative.

^c^FN: false negative.

^d^FP: false positive.

^e^SE: sensitivity.

^f^SP: specificity.

^g^PPV: positive predictive value.

^h^NPV: negative predictive value.

## Discussion

### Principal Results

We found strong agreement between reference standards created through review of EMR free-text encounter notes compared to EMR short diagnostic text fields. Similar to other studies, we also found that when available, free-text encounter notes can capture additional information about a patient for identification of disease, symptoms, and management strategies [7,12,14,15]. Although free-text encounter notes provided additional information regarding risk factors and symptoms, when compared to short diagnostic text fields, their inclusion did not dramatically impact the validation of algorithms intended to identify diagnosed cases. Primary care settings in our sample include regionally or privately operated clinics, different EMR systems, and privacy and confidentiality regulations that can make free-text data difficult to obtain [27]. We found that when free-text encounter notes are unavailable, short diagnostic text data offer a viable option for identification of a confirmed diagnosis among primary care patients, even when this condition is complex such as PTSD.

### Comparison With Prior Work

The estimated PTSD prevalence ranged from 0.8% to 1.3%. Case definition 1, which focused on specific ICD-9-CM code for PTSD (309.81) found a prevalence of 1.1% but may not be viable if 5-digit billing codes (ie, ICD-9-CM) are not available. Within the Manitoba data set, diagnostic code alone and diagnostic codes supplemented with NLP both had high agreement with reference sets. Inclusion of free-text encounter notes during medical record review did not significantly change agreement metrics. Contrary to similar studies, we did not find that the inclusion of NLP improved the agreement of our case definition in Manitoba [7,12,14,15]. However, when we applied the case definitions to the pan-Canadian CPCSSN reference set, provincial differences in diagnostic codes and EMR structure were noticed. Seungwon et al [27] conducted a scoping review of 274 articles representing 299 algorithms for Charlson conditions reporting that case validation studies frequently focused on a single-center, limiting generalizability of created algorithms. Similarly, we found that our algorithm tested in MaPCReN, which includes only 3 distinct EMR venders, performed better than when tested in a pan-Canadian CPCSSN data set representing 11 different EMR venders across Canada.

Consistent with other literature regarding complex phenotypes, we found that reliance on diagnostic codes can vary in accuracy depending on the jurisdiction [14,27]. System-level and jurisdictional differences in diagnostic coding requirements reduced the sensitivity of case definition 1 in the CPCSSN reference set. Depending on the condition, a 3-digit ICD-9-CM code may still indicate disease presence. For example, ICD-9-CM 250 indicates diabetes with ICD-9-CM subcodes indicating the type and severity of the diabetes [28]. However, the 3-digit ICD-9-CM code for PTSD is 309, indicating an adjustment reaction which is not specific to PTSD. When using free-text data to improve PTSD capture, tools such as well-developed and defined NLP or lasso regression can aid in the identification of patients [7,12,14,15]. Case definition 4 supplemented specific diagnostic codes with NLP of short diagnostic text fields in the EMR to identify patients with PTSD. Similar to other works, we found that combining structured EMR data and unstructured free text significantly improved diagnostic capture in our pan-Canadian data set yielding higher performance [7,15,20,27]. However, we did not ascertain additional benefit from using free-text encounter notes when compared to short diagnostic text fields that are more widely available. Doan et al [12] found that NLP showed comparable performance in disease identification to clinician manual chart review. Although literature suggests the need to capture multiple risk factors for the identification of PTSD [19], in this study, we focused NLP on explicit PTSD diagnostic text documented in short diagnostic text fields of the EMR. We demonstrated that explicit PTSD diagnostic text can improve PTSD capture in a pan-Canadian data set. NLP can serve as a model for decision support closing documentation gaps and overcoming barriers present when only structured data fields are available [12,15].

Following free-text encounter note review, 6.1% (20/327) of patients in our purposefully selected reference standard were identified as having “possible PTSD.” These patients did not have an explicit PTSD diagnosis in the text or structured data fields of the EMR. Characterizing patients with “possible PTSD” may identify patients who warrant further clinical investigation to inform diagnosis. Identification of patients with “possible PTSD” can support patient care by informing diagnostic investigations, as well as promoting documentation of mental health symptoms, treatments, and improvements in symptoms [15]. This may be a role for clinical decision support systems that can provide passive alerts to primary care providers indicating the need for further PTSD assessment [7,15].

Depending on study objectives and data set, researchers may choose to use different combinations of coded and free-text data, the former being more readily available and commonly used in many jurisdictions [14,27]. However, previous studies have demonstrated that using diagnostic codes from one part of the EMR alone may be problematic due to data quality concerns [18,29]. Furthermore, changes in terminology and coding standards can make it difficult to compare and share algorithms between EMR systems and jurisdictions. Understanding the health system structure and setting of the study is crucial in algorithm development [27]. Interpretability is an important consideration within the clinical domain, which may suggest the use of an NLP rule-based system, particularly when a data set has limited free-text information. Despite this, the supplementation of structured EMR data with NLP-derived data is important to overcome documentation gaps [9,15,20]. Our pan-Canadian data set only included short diagnostic fields and did not include free-text encounter notes. The availability of free-text encounter notes may suggest the use of a pretrained model for both text representation and classification. Pretrained model such as the Bidirectional Encoder Representations from Transformers can transform free-text data into a standardized form [9]. Specialist models such as MentalBERT have developed domain-specific pretrained language models in the area of mental health that can further benefit machine learning models aimed at capturing mental health conditions [30]. Matching data sets to appropriate methods can balance interpretability of the model and improve prediction leading to results that can inform clinical decision-making and health system planning [9,15,19,20].

### Limitations

This study relied on primary care provider documentation in the EMR. NLP assessment of clinical notes entered by a primary care provider requires processing of clinical narratives that were entered by providers with limited time and may therefore include domain-specific abbreviations and spelling or editorial errors [7]. Due to variation in primary care provider documentation and coding, our study may have underestimated the presence of PTSD in its patient population. Additionally, clinicians primarily use their EMR for clinical purposes and therefore are less concerned with the secondary use of specific ICD-9-CM codes. This may contribute to issues with data capture or completeness. The use of NLP must be developed within context to meet organizational challenges of structured data fields [14]. Tools developed through this study can support identification in a Canadian EMR data repository but have not been validated in other jurisdictions. CPCSSN represents care received from a primary care provider and therefore does not represent care received from a specialist, such as a psychiatrist or psychologist. Future studies linking this data set to other data holdings representing care providing by specialist providers may improve our case definition accuracy by including more dedicated assessments and information related to PTSD care.

### Conclusions

Inclusion of free-text encounter notes during medical record review did not lead to dramatically improved capture of PTSD cases, nor did it lead to significant improvements in case definition agreement. However, incorporating NLP of short diagnostic text fields into a case definition for a complex condition, such as PTSD, improved the capture of our case definition when compared to case definitions that used structured data fields alone. Depending on the jurisdiction and EMR systems in use, specific diagnostic codes can still provide a good estimate of patients with PTSD in a population.

Further research is required to refine NLP algorithms to be able to detect PTSD from free-text encounter notes lacking a formal coded diagnosis entry. In this large primary care data set, PTSD affected between 0.8% and 1.3% of the population, demonstrating that primary care EMR data are a rich source of data for this complex condition.

## Appendix

Multimedia Appendix 1 Data extraction form.

Multimedia Appendix 2 Posttraumatic stress disorder terms.

## Abbreviations

CPCSSN: Canadian Primary Care Sentinel Surveillance Network
EMR: electronic medical record
ICD-9-CM: International Classification of Disease, ninth edition, clinical modification
MaPCReN: Manitoba Primary Care Research Network
NLP: natural language processing
NPV: negative predictive value
PPV: positive predictive value
PTSD: posttraumatic stress disorder
