# Supplementary material for: Pan-Canadian Electronic Medical Record Diagnostic and Unstructured Text Data for Capturing PTSD: Retrospective Observational Study
Source: JMIR Med Inform. 2022 Dec 13;10(12):e41312. doi: 10.2196/41312 (PMC9795397; doi:10.2196/41312)
Supplement: Multimedia Appendix 1 [file medinform_v10i12e41312_app1.pdf]

Appendix A: Data Extraction Form

Patient ID number	Patients with True PTSD	Patient Count (number of records)	Explicit PTSD diagnosis (Y/N)?	≥1 diagnostic criteria of PTSD stated (Y/N)?	diagnosis (key words related to PTSD)	How many criteria does the patient have	Criterion A (Trauma)?	Criterion B (Re-experience)?	Criterion C (Avoidance)?	Criterion D (Distress)?	Criterion E (Hyperarousal)?	Criterion F (Duration > 1 month)?

CONTINUED.

Criterion G (Functional impairment)?	Criterion H (Symptoms unrelated to medication, drug use, or other illness)?	Symptoms (key words related to recorded symptoms)	Military Family	Veteran	Canadian Forces (Yes, No, Unknown)	Comments	
