# Supplementary material for: Pan-Canadian Electronic Medical Record Diagnostic and Unstructured Text Data for Capturing PTSD: Retrospective Observational Study
Source: JMIR Med Inform. 2022 Dec 13;10(12):e41312. doi: 10.2196/41312 (PMC9795397; doi:10.2196/41312)
Supplement: Multimedia Appendix 2 [file medinform_v10i12e41312_app2.pdf]

Appendix B: PTSD terms

Terms for Case definition 4	
Inclusion terms	Unconfirmed PTSD terms *If present requires second PTSD encounter
PTSD	?
ETAT DE STRESS POST-TRAUMAT	LIKELY
POST TRAUMATIC STRESS DISORDER	POSSIBLE
POST TRAUMATIC SD	W PTSD FEATURES
POST TRAUMATIC STRESS	R/O
POST TRAUMATIC STRESS D	VS
POST TRUAMATIC STRES DISORDER	CONSULTATION
DESORDRE DE STRESS POST TRAUMATIQUE	ASSESSMENT
DÉSORDRE DE STRESS POST-TRAUMATIQUE	QUESTIONNAIRE: PTSD
POST-TRAUMATIC STRESS DISORDER	POST TRAUMATIC
ÉTAT DE STRESS POST-TRAUMATIQUE	POST TRAUMATIC FEATURES
ÉTAT STRESS POST-TRAUMATIQUE	POST TRAUMA
POST TRAUMATIC (STRESS DISORDER)	POST-TRAUMATIC STRESS SYMPTOMS
POST*TRAUMATIC STRESS DISORDER	REMISSION
POSTTRAUM STRESS	PREVIOUS PTSD
POST-TRAUMA STRESS DISORDER	PROBABLE
POSTTRAUMATIC - STRESS DISORDER	PROBABLY
POST-TRAUMATIC DISORDER	CHRONIC POST-TRAUMA
POSTTRAUMATIC STRESS	RESOLVED
POST-TRAUMATIC STRESS	PROVISIONAL
POST-TRAUMATIC STRESS DISORDER	SELF-REPORTED
POSTTRAUMATIC STRESS DISORDER	SUSPECTED
PROLONG POSTTRAUM STRESS DISORD	PAST
PROLONG POSTTRAUM STRESS	PARTIEL
P.T.S.D.	
STRESS POST-TRAUMATIAQUE	
STRESS POST-TRAUMATI	
STRESS POST-TRAUMATIQUE	
TROUBLE DE STRESS POST TRAUMATIQUE	
POSTTRAUMATIC STRES DISORDER	
